# The potential role of pneumococcal conjugate vaccine in reducing acute respiratory inflammation in community-acquired pneumococcal pneumonia

**DOI:** 10.1186/s12929-020-00680-9

**Published:** 2020-08-19

**Authors:** Ching-Fen Shen, Shih-Min Wang, Hsin Chi, Yi-Chuan Huang, Li-Min Huang, Yhu-Chering Huang, Hsiao-Chuan Lin, Yu-Huai Ho, Chao A. Hsiung, Ching-Chuan Liu, Taiwan Pediatric Infectious Disease Alliance

**Affiliations:** 1 Department of Pediatrics, National Cheng Kung University Hospital, College of Medicine, National Cheng Kung University, 138, Sheng Li Road, North Dist, Tainan, 70403 Taiwan; 2 Taiwan Pediatric Infectious Disease Alliance, Taipei City, Taiwan; 3 Department of Emergency Medicine, National Cheng Kung University Hospital, College of Medicine, National Cheng Kung University, Tainan City, Taiwan; 4 Department of Pediatrics, Mackay Children’s Hospital, Mackay Medical College, Taipei City, Taiwan; 5 Division of Infectious Diseases, Department of Pediatrics, Kaohsiung Chang Gung Memorial Hospital and Chang Gung University College of Medicine, Kaohsiung City, Taiwan; 6 Department of Pediatrics, National Taiwan University and Hospital, Taipei City, Taiwan; 7 Department of Pediatrics, Chang Gung Memorial Hospital and Chang Gung University College of Medicine, Taoyuan, Taiwan; 8 College of Medicine, China Medical University, Taichung City, Taiwan; 9 Division of Pediatric Infectious Diseases, China Medical University Children’s Hospital, Taichung City, Taiwan; 10 Division of Infectious Disease, Department of Medicine, Buddhist Tzu Chi General Hospital, Hualien, Taiwan; 11 Institute of Population Health Sciences, National Health Research Institutes, Zhunan Township, Taiwan; 12 Center of Infectious Disease and Signaling Research, National Cheng Kung University, Tainan City, Taiwan

**Keywords:** Community-acquired pneumonia, Epidemiology, Pediatric, Pneumococcal conjugate vaccine, Pneumococcal pneumonia, Serotype

## Abstract

**Background:**

Pneumococcal conjugate vaccine (PCV) reduces both invasive pneumococcal disease (IPD) and other pneumococcal infections worldwide. We investigated the impact of stepwise implementation of childhood PCV programs on the prevalence of pneumococcal pneumonia, severity of acute inflammation, and associations between breakthrough pneumonia and pneumococcal serotypes in Taiwan.

**Methods:**

In total, 983 children diagnosed with community-acquired pneumococcal pneumonia were enrolled between January 2010 and December 2015.

**Results:**

Proportions of pneumococcal vaccinations increased each year in age-stratified groups with PCV7 (32.2%) as the majority, followed by PCV13 (12.2%). The proportion of pneumococcal pneumonia decreased each year in age-stratified groups, especially in 2–5 year group. Serotype 19A is the leading serotype either in vaccinated (6.4%) or unvaccinated patients (5.2%). In particular, vaccinated patients had significantly higher lowest WBC, lower neutrophils, lower lymphocytes and lower CRP values than non-vaccinated patients (*p* < 0.05). After stratifying patients by breakthrough infection, those with breakthrough pneumococcal infection with vaccine coverage serotypes had more severe pneumonia disease (p < 0.05).

**Conclusion:**

Systematic childhood pneumococcal vaccination reduced the prevalence of community-acquired pneumococcal pneumonia, especially in 2–5 year group. Serotype 19A was the major serotype for all vaccine types in patients with pneumococcal pneumonia and severity of acute inflammatory response was reduced in vaccinated patients.

## Background

Bacteremic pneumococcal pneumonia is a major global health problem with a deleterious impact on patients’ lives, presenting a management challenge to clinicians because of its complications, especially in children and older adults [1, 2]. Pneumococcal disease is the primary cause of childhood morbidity and mortality worldwide, resulting in 0.7 to 1.0 million deaths each year in children younger than age 5 years [3]. Childhood community-acquired pneumonia is most often attributed to *Streptococcus pneumoniae*, and the greatest incidence of invasive pneumococcal infection (IPD) is observed in children younger than age 2 [4]. Many countries have had reductions in IPDs since 2000 after the introduction of pneumococcal conjugate vaccine (PCV), specifically 7-valent conjugate pneumococcal vaccine (PCV7) containing seven serotypes [5–8]. Universal vaccination with PCV7 also resulted in a 77% reduction in pneumococcal disease in children aged 1–5 and a 39% reduction in hospitalization of children age under age 2 [3]. A recent review and meta-analysis reported that systematic childhood PCV immunization programs provided widespread protection against IPD and reduced cases by 90% within 10 years [9].

Taiwan began an IPD surveillance program in October 2007, requiring every hospital to report IPD cases of all ages and to send *S. pneumoniae* isolates to the Taiwan Centers for Disease Control (Taiwan CDC) [10]. The highest burden of IPD in Taiwan occurs in children younger than age 5, particularly those aged 2–4 years, among whom the incidence was 21.1 per 100,000 population per year between 2008 and 2012 [10]. The serotype 19A became most prevalent in 2010 and was the dominant IPD serotype in Taiwanese infants and children between 2008 and 2014 [10, 11]. During July 2009 and February 2013, the Taiwan CDC introduced a series of public-funded PCV programs targeting different groups of children at high-risk for IPD, including children younger than 5 years of age with certain medical conditions susceptible to IPD, or those who were born into families with low or middle income. A nationwide stepwise catch-up program for 13-valent PCV (PCV13) was then launched in March 2013, targeting children aged 2–5 years [12]; the age-range was then expanded in 2014 to cover children aged 1–5 years, and a systematic PCV program introduced in January 2015 provided routine two-plus-one immunization for all infants [13].

Despite early and appropriate antibiotic treatment, mortality associated with community-acquired pneumonia (CAP) still remains high [14], especially in patients with underlying clinical illness. Approximately 18% of patients hospitalized for CAP match the criteria for severe CAP, with more patients presenting with septic shock and need for mechanical ventilation. The mortality rate of severe CAP can be as high as 29% [15]. Progression to septic shock is believed to be caused by excessive or uncontrolled local and systemic pro-inflammatory response [16]. A stronger inflammatory response is associated with treatment failure and mortality [17].

Specific pneumococcal serotypes are associated with different clinical patterns of pneumococcal disease, classified according to their capacity to cause invasive disease [18]. Serotypes 1, 5 and 7F are highly invasive serotypes associated with invasive disease in younger adults, but with low mortality rates. In contrast, serotypes 3, 19F and 23F possess relatively low invasive potential, affecting older adult patients with comorbidities and increasing case-fatality rates [18–21]. Among all pneumococcal disease, pneumococcal pneumonia serotypes are associated with the greatest severity and highest mortality rates [22, 23]. However, to the best of our knowledge, no previous studies have addressed the relationship between PCV, acute inflammation, respiratory failure, and associations with pneumococcal serotypes. Therefore, the overall purpose of the present study was to investigate the impact of stepwise implementation of pneumococcal conjugate vaccine (PCV) on epidemiological change in pneumococcal CAP, including: 1) potential reduction in the prevalence of pneumococcal pneumonia, 2) whether or not childhood PCV vaccination affects the severity of acute inflammation in those who develop pneumococcal pneumonia, and 3) whether breakthrough pneumococcal pneumonia is associated with specific pneumococcal serotypes or not.

## Materials and methods

### Study design and sample recruitment

Children and adolescents under 18 years of age who had been diagnosed with CAP were recruited prospectively between January 2010 and December 2015 from the Taiwan Pediatric Infectious Diseases Alliance (TPIDA), a collaborative consortium of pediatric institutes in nine major medical centers in Taiwan. TPIDA contained pediatric infectious disease departments of tertiary medical centers, including National Taiwan University Hospital (Taipei City, Taiwan); Mackay Memorial Hospital (Taipei City, Taiwan); Chang Gung Memorial Hospital (Linkou, Taiwan); China Medical University Hospital (Taichung City, Taiwan); National Cheng Kung University Hospital (Tainan City, Taiwan), Kaohsiung Chang Gung Memorial Hospital (Kaohsiung City, Taiwan), and Buddhist Tzu Chi General Hospital (Hualien, Taiwan). TPIDA conducted nationwide surveillance of childhood CAP from Jan. 2010 to Jan. 2016. CAP was defined as acute lung parenchymal change and pulmonary infiltrates on chest X-ray with associated clinical symptoms or sign of respiratory tract infection. Patients’ demographic data, clinical manifestations and chest radiographic characteristics were recorded.

### Ethical considerations

The protocol of this study was reviewed and approved by the Institutional Review Board (IRB) of each hospital in TPIDA, and was also approved by the IRB of National Cheng Kung University Hospital (NCKU) (No. HR-98-112). Signed informed consent to participate in the study was obtained from each included patient or the patient’s parents or guardian.

### Specimen and pathogen identification

Multiplex PCR of pleural effusion was performed to identify respiratory bacterial pathogens, including *S. pneumoniae*, *Moraxella catarrhalis*, *Haemophilus influenzae*, *Staphylococcus aureus*. Serum samples were tested for the presence of antibodies to *M. pneumoniae* by using the IgM-specific Mycoplasma Immuno-Card, an enzyme immunoassay from Meridian Bioscience (Cincinnati, OH, USA), and the *M. pneumoniae* IgG/IgM Antibody Test System (FTI-SERODIA-myco II test; Fujirebio Inc., Taipei, Taiwan) following the manufacturers’ instructions. The serotypes of pneumococcal isolates were identified by a multiplex polymerase chain reaction (PCR) method as described in previous studies [24, 25]. Nineteen different serotypes were determined by 5 sequential multiplex PCR reactions: reaction 1 includes serotypes 6A/B, 9 V, 15B/C, 18C, 19F, reaction 2 includes serotypes 3, 14, 19A, 23F, reaction 3 includes1, 4, 5, 23A, reaction 4 includes15A, 7F, 22F and reaction 5 includes serotype 20, 34, 33F. The serotypes not included in the multiplex PCR reactions were defined as non-typed serotypes.

Pneumococcal pneumonia was divided into two groups by the following definitions [2]. Definitive pneumococcal pneumonia (133 patients) was defined by either *S. pneumoniae* isolated from sterile body sites, major blood or pleural effusion, or positive pneumococcal PCR [24, 26] from pleural effusion [1]. Probable pneumococcal pneumonia (850 patients) was defined by positive urine or pleural effusion pneumococcal antigen reaction without isolating pneumococci or positive PCR result.

### Statistical analysis

Descriptive analyses of numerical variables are presented as mean and standard deviation (mean ± SD), and categorical variables are presented as frequency and percentage. Student’s t test was used for analyzing normal distribution of continuous variables between groups; Mann-Whitney U test was used for un-normal distribution of continuous variables between groups. Chi-square test and Fisher’s exact test were used for analyzing categorical variables. Logistic regression analysis was used to evaluate associations between definitive pneumococcal pneumonia and vaccine type. All test hypotheses were evaluated using two-tailed t tests. A *P*-value of< 0.05 was considered statistical significance. All statistical analyses were performed using SAS 9.4 software (SAS Institute Inc., Cary, NC, USA.).

## Results

### Patients’ characteristics

**Fig. 1:**
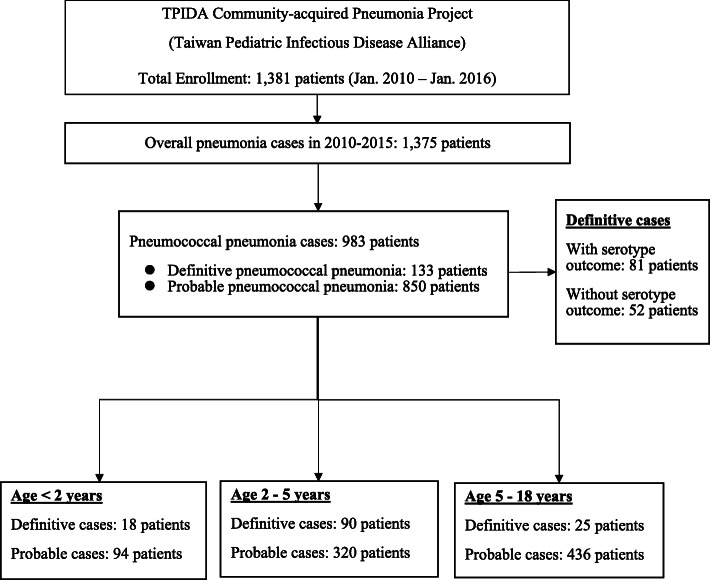
Flowchart of pneumococcal pneumonia patient selection from TPIDA pneumonia project

**Fig. 2:**
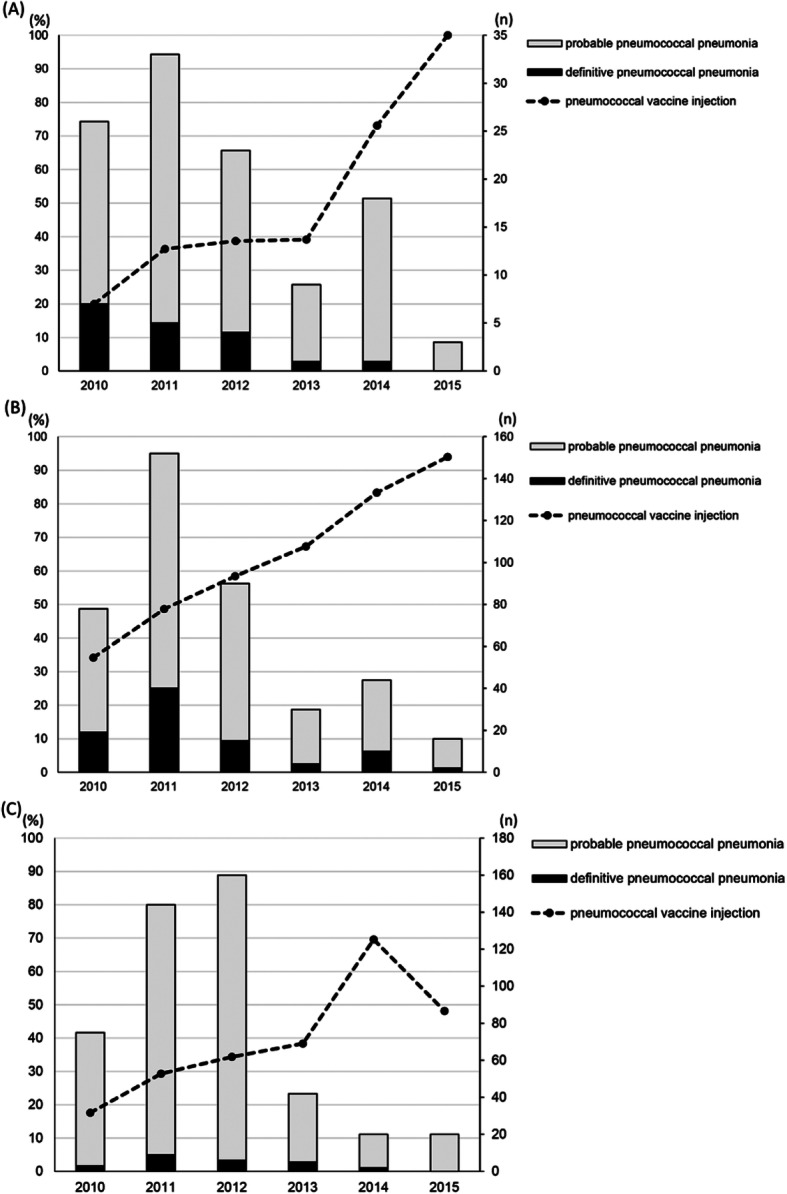
Proportions of pneumococcal pneumonia (probable and definitive) among all type of pneumonia and the proportion of cases receiving any pneumococcal vaccination during 2010-2015. (**a**) Age<2 years (**b**) Age: 2-5 years (**c**) Age: 5-18 years

The flowchart of study sample recruitment is shown in Fig. 1. A total of 1375 subjects were enrolled between 2010 and 2015, of whom 983 subjects fulfilled the criteria of pneumococcal pneumonia and were hospitalized for treatment. The prevalence of pneumococcal pneumonia among all-cause pneumonia fluctuated between years, with the lowest prevalence in 2015 (2010: 72.8%; 2011: 74.7%; 2012: 72.4%; 2013: 58.7%; 2014: 79.6%; 2015: 54.9%) shown in Table 1. However, cases of all-cause pneumonia and pneumococcal pneumonia have decreased dramatically since 2013 and have continued to decrease in subsequent years. The overall proportion of pneumococcal vaccinations in this pneumonia cohort increased incrementally year by year, from 25.1 to 66.7% (2010: 25.1%; 2011: 38.0%; 2012: 41.8%; 2013: 55.6%; 2014: 80.5%; 2015: 66.7%) (Table 2). After stratifying by age groups (< 2 years, 2–5 years and 5–18 years), the proportion of pneumococcal vaccinations increased year by year in each group (Table 2). The proportions of vaccinations increased more in children age under 5 years old compared with those age 5–18 years (Table 2 and Fig. 2). The proportion of pneumococcal pneumonia decreased in the 2–5 years group from 2010 to 2012 compared with the proportion in the < 2 years or 5–18 years groups (Fig. 2). The distribution of different types of pneumococcal vaccination by year is shown in Table 2. Of note, 4 patients received PCV10 vaccine and 1 received PPV23 in 2014; and 3 patients received PCV10 in 2015. However, these 7 patients who received PCV10 conjugate vaccine were given PCV13 boosters after 2014. PCV7 is the major type of pneumococcal vaccine (32.2%) among all vaccinated patients, followed by PCV13 (12.2%). PCV7 was predominant during 2012 ~ 2013 (91.2 to 95.6%), but it was replaced by PCV13 after 2013 (48.9% in 2013, 81.8% in 2014, 100% in 2015). This clearly demonstrates the impact of national stepwise implementation of PCV13 into the routine childhood vaccination schedule. The distribution of serotypes was similar between vaccinated patients and non-vaccinated patients with pneumococcal pneumonia (Table 3). Serotype 19A is the leading serotype either in vaccinated (6.4%) or unvaccinated patients (5.2%), followed by serotype 3 (vaccinated: 1.7%; unvaccinated: 2.1%).

**Table 1:** Yearly distribution of all-cause pneumonia, definitive and probable pneumococcal pneumonia cases during 2010~2015

	All-cause pneumonia cases	Pneumococcal pneumonia cases		
Year	N	Total	Definitive	Probable
2010	246	179 (72.8)	29 (11.8)	150 (61.0)
2011	440	329 (74.7)	54 (12.3)	275 (62.5)
2012	377	273 (72.4)	25 (6.6)	248 (65.7)
2013	138	81 (58.7)	10 (7.2)	71 (51.5)
2014	103	82 (79.6)	13 (12.6)	69 (67.0)
2015	71	39 (54.9)	2 (2.8)	37 (52.1)
Total	1375	983 (71.5)	133 (9.7)	850 (61.8)

Data are shown as N (%)

**Table 2:** Yearly vaccination status according to age group and type of pneumococcal vaccine received during 2010 ~ 2015

	Pneumococcal pneumonia cases by year						
	2010 (*n* = 179)	2011 (*n* = 329)	2012 (*n* = 273)	2013 (*n* = 81)	2014 (*n* = 82)	2015 (*n* = 39)	Total (*n* = 983)
Cases with any pneumococcal vaccination according to age	45 (25.1)	125 (38.0)	114 (41.8)	45 (55.6)	66 (80.5)	26 (66.7)	421/983 (42.8)
< 2 years	5/26 (19.2)	9/33 (27.3)	9/23 (39.1)	3/9 (33.3)	13/18 (72.2)	3/3 (100)	42/112 (37.5)
2-5 years	27/78 (34.6)	78/152 (51.3)	55/90 (61.1)	26/30 (86.7)	39/44 (88.6)	15/16 (93.8)	240/410 (65.9)
5-18 years	13/75 (17.3)	38/144 (26.3)	50/160 (31.3)	16/42 (38.1)	14/20 (70.0)	8/20 (40.0)	139/461 (30.2)
Type of pneumococcal vaccine received							
PCV7	43/45 (95.6)	116/125 (92.8)	104/114 (91.2)	32/45 (71.1)	21/66 (31.8)	0/26 (0.0)	316/983 (32.2)
PCV10	0 (0.0)	3/125 (2.4)	2/114 (1.75)	3/45 (6.7)	4/66 (6.1)	3/26 (11.5)	15/983 (1.5)
PCV13	1/45 (2.2)	2/125 (1.6)	15/114 (13.2)	22/45 (48.9)	54/66 (81.8)	26/26 (100.0)	120/983 (12.2)
PPV23	1/45 (2.22)	5/125 (4.0)	1/114 (0.9)	0/45 (0.0)	1/66 (1.5)	0/26 (0.0)	8/983 (0.8)

Data are shown as n/N (%)

PCV7, 7-valent polysaccharide conjugate vaccine; PCV10, 10-valent polysaccharide conjugate vaccine; PCV13, 13-valent polysaccharide conjugate vaccine; PPV23, 23-valent polysaccharide pneumococcal vaccine

**Table 3:** Pneumococcal serotypes, clinical characters and laboratory findings of children with pneumococcal pneumonia according to vaccination status and type of vaccine received

	Received pneumococcal vaccine		*p*-value^b^	Received pneumococcal vaccine	
	No	Yes		PCV7	PCV 13
	*N* = 562	*N* = 421		*N* = 316	*N* = 120
Probable pneumococcal pneumonia	487 (86.6)	363 (86.2)		274 (86.7)	104 (86.7)
Definitive pneumococcal pneumonia	75 (13.4)	58 (13.8)	0.845	42 (13.3)	16 (13.3)
Serotype^--^					
Type 3	12 (2.1)	7 (1.7)	0.594	6 (1.9)	1 (0.8)
Type 6	1 (0.2)	1 (0.2)	1.000	0	1 (0.8)
Type 14	1 (0.2)	1 (0.2)	1.000	0	1 (0.8)
Type 19A	29 (5.2)	27 (6.4)	0.402	22 (7.0)	6 (5.0)
Type 19F	1 (0.2)	2 (0.5)	0.580	1 (0.3)	1 (0.8)
Unknown	518 (91.1)	383 (90.9)	0.502	287 (90.8)	110 (91.7)
**Processing of pneumonia severity**					
Pleural fluid aspiration	61 (10.9)	38 (9.0)	0.346	27 (8.5)	10 (8.3)
Chest catheter	86 (15.3)	58 (13.8)	0.503	45 (14.2)	13 (10.8)
VATS	54 (9.6)	43 (10.2)	0.753	31 (9.8)	12 (10.0)
ICU admission	132 (23.5)	946 (22.3)	0.669	71 (22.5)	22 (18.3)
Days in ICU	9.6 ± 11.0	7.2 ± 8.1	0.058^c^	7.1 ± 8.4	7.1 ± 7.5
O2 use	195 (34.7)	155 (36.8)	0.492	108 (34.2)	46 (38.3)
O2 use day >=7 days	60 (10.7)	49 (11.6)	0.634	37 (11.7)	12 (10.0)
Admission days >= 14 days	95 (16.9)	69 (16.4)	0.831	52 (16.5)	16 (13.3)
**Blood tests**					
Lowest Hb (g/ dl)	11.6 ± 3.7	11.4 ± 1.5	0.289^c^	11.5 ± 1.4	11.4 ± 1.6
Highest WBC (x k/ cumm)	14.0 ± 9.1	13.9 ± 8.3	0.832^c^	13.8 ± 8.7	14.1 ± 7.6
Lowest WBC (x k/ cumm)	8.8 ± 4.8	9.5 ± 5.7	**0.026**^**c**^	9.5 ± 6.0	9.9 ± 5.3 ^a^
Highest Bandemia (%)	7.2 ± 11.8	6.1 ± 10.2	0.128^c^	6.2 ± 10.7	5.4 ± 8.2 ^a^
Highest Neutrophils (x k/ cumm)	34.0 ± 32.3	27.9 ± 29.9	**0.003**^**c**^	33.3 ± 32.1	12.2 ± 14.0 ^a^
Highest Lymphocytes (x k/ cumm)	12.5 ± 15.5	10.2 ± 14.8	**0.021**^**c**^	12.6 ± 16.0	3.3 ± 7.3 ^a^
Highest Eosinophils (x k/ cumm)	0.9 ± 2.0	1.2 ± 3.4	0.083^c^	0.9 ± 1.7	2.2 ± 5.7 ^a^
Highest Platelets (x k/ cumm)	368.1 ± 200.9	366.2 ± 196.5	0.883^c^	360.0 ± 199.1	377.1 ± 194.6
Highest CRP (mg/ dl)	13.2 ± 12.4	10.9 ± 11.0	**0.002**^**c**^	11.5 ± 11.4 ^a^	8.9 ± 9.5 ^a^

^--^ defined by PCR results

Data are shown as N (%) for categorical variable and mean ± standard deviation for continuous variable, VATS, video-assisted thoracoscopic surgery; ICU, intensive care unit

Numbers in bold indicate statistical significance (p<0.05)

^a^ significantly different from non-vaccination group

^b^ Chi-square test

^c^ Student's t-test

### Clinical outcomes

Univariate analysis (Table 3) revealed that pleural fluid aspiration, chest catheter use, ICU admission, and days in ICU were lower in vaccinated patients compared to non-vaccinated patients, but the difference was not statistically significant (*p* > 0.05). However, vaccinated patients had significantly higher lowest WBC, lower neutrophils, lower lymphocytes and lower CRP values than non-vaccinated patients (all *p* value < 0.05). When looking into the specific pneumococcal vaccines received, patients receiving either PCV7 or PCV13 had significantly lower CR*P* values than those who did not receive any pneumococcal vaccine, while patients receiving PCV13 had the lowest CRP value (8.9 ± 9.5 mg/dl vs 13.2 ± 12.4 mg/dl in non-vaccinated patients, *p* value = 0.002). Patients who received PCV13 pneumococcal vaccine had significantly higher WBC, lower bandemia, lower neutrophils, lower lymphocytes, higher eosinophils and lower CRP values than those who did not receive any pneumococcal vaccine (all *p* value < 0.05).

Patients who received any pneumococcal vaccine and had definitive pneumococcal pneumonia were divided into subgroups of vaccine coverage breakthrough infection and non-vaccine type infection according to pneumococcal serotypes (Table 4). Those with breakthrough pneumococcal infection with vaccine coverage serotypes had more severe pneumonia, including higher risk of chest catheter use, video-assisted thoracoscopic surgery, and any one to three complications compared with non-vaccine serotypes (all *p* value < 0.05). Breakthrough infection with vaccine coverage serotypes also presented with lower Hb level, higher eosinophils, and higher CRP than the non-vaccine serotype group (all *p* value< 0.05).

**Table 4:** Comparison of clinical characters of non-vaccine serotypes and vaccine serotypes related definite pneumococcal pneumonia

	Coverage serotype		*p*-value
	No^c^	Yes^d^	
	*N* = 21	*N* = 37	
**Processing of pneumonia severity**			
Pleural fluid aspiration	7 (33.33)	19 (51.35)	0.185^a^
Chest catheter use	12 (57.14)	31 (83.78)	**0.026**^**a**^
VATS	9 (42.86)	29 (78.38)	**0.006**^**a**^
ICU admission	12 (57.14)	30 (81.08)	0.050^a^
Days in ICU	6.9 ± 4.1	8.6 ± 11.9	0.635^b^
O2 use	14 (66.67)	30 (81.08)	0.218^a^
O2 use day >=7 days	8 (38.10)	16 (43.24)	0.702^a^
Admission days >= 14 days	12 (57.14)	29 (78.38)	0.088^a^
One of the above	16 (76.19)	37 (91.38)	**0.004**^**a**^
Two of the above	14 (66.67)	36 (97.30)	**0.002**^**a**^
Three of the above	14 (66.67)	34 (91.89)	**0.027**^**a**^
**Blood tests**			
Lowest Hb (g/ dl)	10.0 ± 1.8	9.2 ± 0.9	**0.049**^**b**^
Highest WBC (x k/ cumm)	19.6 ± 9.2	24.1 ± 9.3	0.080^b^
Highest Bandemia (%)	6.9 ± 9.1	11.6 ± 17.0	0.198^b^
Highest Neutrophilis (x k/ cumm)	39.1 ± 31.6	55.6 ± 32.4	0.069^b^
Highest Lymphocytes (x k/ cumm)	11.9 ± 14.1	18.4 ± 16.5	0.132^b^
Highest Eosinophils (x k/ cumm)	0.9 ± 1.8	2.2 ± 2.4	**0.038**^**b**^
Highest Platelets (x k/ cumm)	577.8 ± 228.9	663.4 ± 249.0	0.201^b^
Highest CRP (mg/ dl)	23.7 ± 10.6	29.5 ± 9.8	**0.038**^**b**^

Numbers in bold indicate statistical significance (*p*<0.05)

Data are shown as N (%) for categorical variable and mean ± standard deviation for continuous variable; VATS, video-assisted thoracoscopic surgery; ICU, intensive care unit

^a^ Chi-square test

^b^ Student's t-test

^c^ all of the non-vaccine coverage serotypes are unknown serotypes

^d^ vaccine coverage serotype including 7 with serotype 3, one case with serotype 6, 27 with serotype 19A, 2 with serotype 19F

## Discussion

Results of this study clearly demonstrated that implementing a systematic immunization program to administer pneumococcal vaccine to infants and children reduces the prevalence of pneumococcal pneumonia in the community. During the study period, serotype 19A was the major serotype in patients with pneumococcal pneumonia who had received any type of vaccine in Taiwan. Serotypes are a crucial element in determining the severity of pneumonia and inflammation in patients with pneumococcal pneumonia. The severity of acute inflammatory response was reduced in vaccinated patients with pneumococcal pneumonia compared with that in unvaccinated patients. To date, our study is the first report discussing the attenuation effect on inflammatory response in breakthrough pneumococcal infection. Other than pneumococcal infection, the influenza vaccine had been reported to reduce symptoms severity among patients with influenza A/H3N2 disease (vaccine failures) [27]. Hence, in the present study, the evidence of vaccine reducing the systemic inflammation in breakthrough IPD still supports the universal use of PCV13 in children, providing additional value regardless of vaccine failure.

Results of previous studies are largely compatible with results of the present study. A national case-control study of the effectiveness of PCV against IPD (conducted by the Taiwan CDC) demonstrated a vaccine effectiveness of 80% for an age-appropriate PCV13 program [28]. Also, in the same program, effectiveness against serotype 19A was 89%, while effectiveness of at least 1 dose of PCV, including either PCV7, PCV10, or PCV13, declined from 81% within 6 months of the last dose of PCV to 19% after 2 years. This indicates that protection was decreasing over time, either because of the waning protective antibody or vaccine serotypes gradually being replaced by other non-vaccine serotypes. However, the effectiveness of catch-up programs is evidenced primarily by changes in IPD incidence over time at the population level, but not at the individual level [29, 30]. Similarly, high effectiveness was shown against vaccine-type IPD in a randomized controlled trial of PCV7 efficacy in American-Indian children when the first PCV dose was given at < 7 months of age and as a catch-up after 12–23 months [31]. However, the modifying effect of age may be a concern for vaccine effectiveness analysis [32]. In addition to IPD, some reports had addressed the influence of pneumococcal vaccine on non-IPD pneumococcal infection, especially pneumonia and otitis media. Conjugate pneumococcal vaccination (either PCV7 or PCV13) had significantly reduced pneumonia in children with congenital heart disease in Mexico (vaccinated, 13.2% vs. unvaccinated 33.6%, *p* < 0.001) [32]*.* In the United States, pneumococcal pneumonia requiring hospitalization significantly decreased in children after PCV13 introduction. Although complicated pneumococcal pneumonia had decreased steadily after PCV13 introduction, the need for intensive care, mechanical ventilation, and invasive procedure remained unchanged. Among those with breakthrough pneumococcal pneumonia, PCV13 serotypes 19A and 3 were still responsible for half of cases in the post-vaccine era [33]. Recent meta-analysis also indicated that PCV10 and PCV13 had a significant impact in reducing hospitalizations for pneumonia, particularly in children aged < 24 months and for radiologically confirmed disease. In children younger than 24 months, the reduction in hospitalization rates was 17% (95%CI: 11–22%, *p*-value< 0.001) and 31% (95%CI: 26–35%, *p* < 0.001) for clinically and radiologically confirmed pneumonia, respectively, after the introduction of PCVs [34]. In the present study, a protective effect was observed in a catch-up program targeting children aged 2 to 5 years starting from 2013 with a stepwise national catch-up immunization program.

Although the proportion of pneumococcal pneumonia among all-cause pneumonia declined in 2013, it resurged in 2014, then declined further to 50% in 2015. The case numbers of all-cause pneumonia and pneumococcal pneumonia indicate that both of these diseases have been decreasing since 2013. However, vaccine use in Taiwan prior to 2013 was different. Public-funded PCV7 vaccine was administered mainly to children ≤5 years of age with underlying diseases or low socioeconomic status between 2009 and 2011. While during 2010 and 2011, public-funded PCV10 was administered mainly to children in mountainous areas or off-shore islands. Then, after 2012, public-funded PCV for vulnerable groups was shifted to PCV13. This change in policy was made based on concerns about local pneumococcal sero-epidemiology and cost effectiveness. Lai et al. reported that the overall vaccine coverage rates for IPD during the period 2000–2012 were 65.2% for PCV7, 65.9% for PCV10, 85.8% for PCV13, and 91.2% for PPV-23. The coverage rates for PCV-7 and PCV-10 decreased significantly with time, but the PCV13 coverage rates remained stable [35]. Public-funded PCV10 was only available to children in mountainous areas for short time and the use in private markets was also much less compared to the use of PCV7 and PCV13. Vaccine use is much different in Taiwan from the universal use of PCV10 in other countries such as Canada, and explains why only 7 patients with previous PCV10 vaccination are included in the present study [36, 37].

Our study also clearly demonstrate that the use of PCV 7 has shifted to PCV13 since 2013, especially in children younger than age 5 years. PCV7 was the most common pneumococcal vaccine used in children (above 90%) before 2013, but was completely replaced by PCV13 in 2015. At the same time, the vaccine coverage rate reached 100% in children younger than age 2 years, and 93.8% in children aged 2 ~ 5 years. Since the incidence of community acquired pneumonia is highest among children younger than 5 years, the implementation of the vaccine program was apparently aimed at the target population. Results of the present study have shown that this vaccine policy not only decreased invasive pneumococcal disease (IPD), but also decreased non-invasive pneumococcal disease (esp. pneumococcal pneumonia). As for the surge of pneumococcal pneumonia cases in 2014, several things need to be clarified. First, according to the CDC report conducted by Wei et al. [35], the IPD incidence decreased significantly in the 2–5 years old group, from 22.8 cases per 100, 000 person-years in 2011–2012 to 11.9 per 100, 000 person-year in 2013. The IPD incidence decreased from 11.4 cases per 100, 000 in 2013 to 7.1 cases per 100, 000 person-years among children 1 year of age in 2014. The reduction in cases contributed majorly to the decrease of serotype 19A. This reveals that the stepwise introduction helps to decrease the incidence of IPD among target age groups [38]. However, in the study conducted by Cho et al. [36], pneumococcal isolates were collected, including both invasive and non-invasive strains, during 2010 ~ 2015 in northern Taiwan, and the number of pneumococcal isolates in 2014 was higher than those in 2013 or 2015. Looking more closely at cases in 2014, most of the pneumococcal isolates came from non-IPD cases (89.4%) [39]. Therefore, we may conclude that the implementation of PCV13 stepwise vaccination policy decreased both IPD and non-invasive pneumococcal disease. Nevertheless, the secular trend of overall pneumococcal disease, including both invasive and non-invasive disease, may fluctuate during the transitional period.

In the present study, patients receiving more types of PCV appears to have more of an impact on reducing the prevalence of pneumococcal pneumonia than patients receiving only one type of vaccine (Supplementary Fig. 1). Also, the major vaccine-types received by patients with pneumococcal pneumonia were PCV 7 and PCV13 (Table 2). A possible explanation for these findings may be that non-PCV7/PCV10-covered serotypes, such as 19A, emerged gradually and that protection against pneumococcal pneumonia may decline with the changing epidemiology of nonvaccine serotypes. Other authors have suggested that this epidemiological trend may influence estimations of vaccine effectiveness [32, 33]. Therefore, children who received more types of PCV appear to increase the covered serotypes (such as 19A), resulting in lower prevalence of pneumococcal pneumonia. PCV13 was found to provide significant protection for most vaccine serotypes, but serotype-specific correlates of protection still appear to vary widely [34]. Associations between serotype-specific IgG concentrations after PCV priming during infancy and long-term clinical protection against vaccine serotypes and non-vaccine serotypes requires further study.

In the present series, an acute inflammation was found in children infected by serotypes contained in PCV13, represented mainly by serotypes 19A and 3. These findings are similar to those of previous studies that describe how serotypes 19A, 3, and 19F were independently associated with respiratory symptoms, including the development of hypoxemic respiratory failure, multi-lobar involvement and the need for mechanical ventilation in adults [38]. However, to the best of our knowledge, no previous studies have addressed the relationship between pneumococcal vaccine and pulmonary or systemic inflammation. Our results show that serotypes 19A and 3 were the main serotypes of all vaccine types, and that the severity of systemic inflammation was reduced in patients receiving PCV13 more than in those receiving PCV7. However, the specific serotypes associated with development of respiratory failure do not have a well-defined pathophysiological explanation. Other studies have shown that the features of the polysaccharide capsule of pneumococcus appear to foster higher resistance to neutrophil activity in the lungs and this allows bacterial growth in the lungs to persist; consequently, the result is prolonged and greater immune response and lung injury are characteristic of serotypes 19A, 3, and 19F [23, 38–40]. Moreover, based on the adhesions they express, the various pneumococcus serotypes differ in their ability to adhere to the respiratory epithelium, resulting in different levels of virulence of serotype-dependent strains [38, 41]. Experimental studies in animal models also have shown that pneumococcal serotypes differ in properties such as resistance to phagocytosis, ability to penetrate into tissues and capacity to activate the inflammatory response [42]. Taken together, the results of these studies provide strong evidence supporting our finding that PCV vaccines may reduce the severity of acute inflammation in children with pneumococcal pneumonia attributed to serotypes 19A, 3, and 19F.

Results of the present study show that patients who received pneumococcal vaccine have higher lowest WBC, and lower neutrophil counts and CRP levels compared to non-vaccinated patients, and this difference was even more prominent in those receiving PCV13 vaccine. This is likely due to the fact that PCV works by eliciting an IgG1 (and IgG3) response to polysaccharide. Meanwhile, it also triggers a stronger immune response because polysaccharides co-expressed with carrier proteins interact better with major histocompatibility complex and mediate cognate CD4+ T-cell help for polysaccharide-specific B-cell activation. This mechanism enhances immunogenicity and supports more rapid and robust production of antibodies and the establishment of T-cell–dependent immunological memory [43]. As for the differences in WBC and neutrophils between vaccinated and non-vaccinated patients observed in the present study, we speculate that this could be explained by the modulated immune response after vaccination. In previous studies, relative leukopenia was found to be a major risk factor for mortality in pneumococcal bacteremia [44]. Actually, in fulminant pneumococcal infection, the host fails to evoke adequate immune response to contain the bacteria, even after producing more neutrophils for phagocytosis, but the total white cell counts are still rapidly consumed. In the present study, we demonstrated that with prior pneumococcal immunization, the vaccinated hosts primed with pre-existing antibody and cellular immunity responded better to breakthrough infection.

This study has several limitations. First, there is a small number of patients who initially received PPV10 or PPV23 vaccination (3 in 2014, and 4 in 2015) and subsequently, received 1–3 doses of PCV13 boosters. However, we have justified that this was due to the change in public funding policy, and further, the proportion was very minor relative to those who received PCV7 or PCV13 vaccine; therefore the influence of prior PPV10 or PPV23 vaccination on the current analysis was considered very limited. Also, we could not evaluate the vaccine effectiveness of each PCV because a larger number of normal healthy children would be needed as a control group for comparison. Thirdly, subjects in this study were actively recruited from medical center patients with pneumonia, so results for vaccine effectiveness are necessarily limited. The recruitment of more severe cases and not including all pneumonia cases may also represent selection bias. Future studies must broaden the sources of pneumonia cases to enhance the reference of research results.

## Conclusion

The present study confirms that routine childhood pneumococcal vaccination reduces the prevalence of community-acquired pneumococcal pneumonia. Results also confirmed that pneumococcus serotypes are associated with the severity of pneumonia and systemic inflammation in patients with pneumococcal pneumonia. Serotype 19A was the major serotype in patients with pneumococcal pneumonia during the study period. Interestingly, the degree of acute systemic inflammatory response was much more reduced in vaccinated patients than unvaccinated patients.

## Supplementary information

**Additional file 1: Supplementary Fig. 1**. Propotions of any type of pneumococcal vaccinations among children with all-cause pneumonia hospitalized from 2010 to 2015. (A) 1 type of vaccine (B) 2 types of vaccine (C) 3 or more types of vaccine.

## Data Availability

The data used to support the findings of this study are included within the article.
