# Supplementary material for: The potential role of pneumococcal conjugate vaccine in reducing acute respiratory inflammation in community-acquired pneumococcal pneumonia
Source: J Biomed Sci. 2020 Aug 19;27:88. doi: 10.1186/s12929-020-00680-9 (PMC7435222; doi:10.1186/s12929-020-00680-9)
Supplement: Supplementary file 1 — Additional file 1: Supplementary Fig. 1. Propotions of any type of pneumococcal vaccinations among children with all-cause pneumonia hospitalized from 2010 to 2015. (A) 1 type of vaccine (B) 2 types of vaccine (C) 3 or more types of vaccine. [file 12929_2020_680_MOESM1_ESM.docx]

**
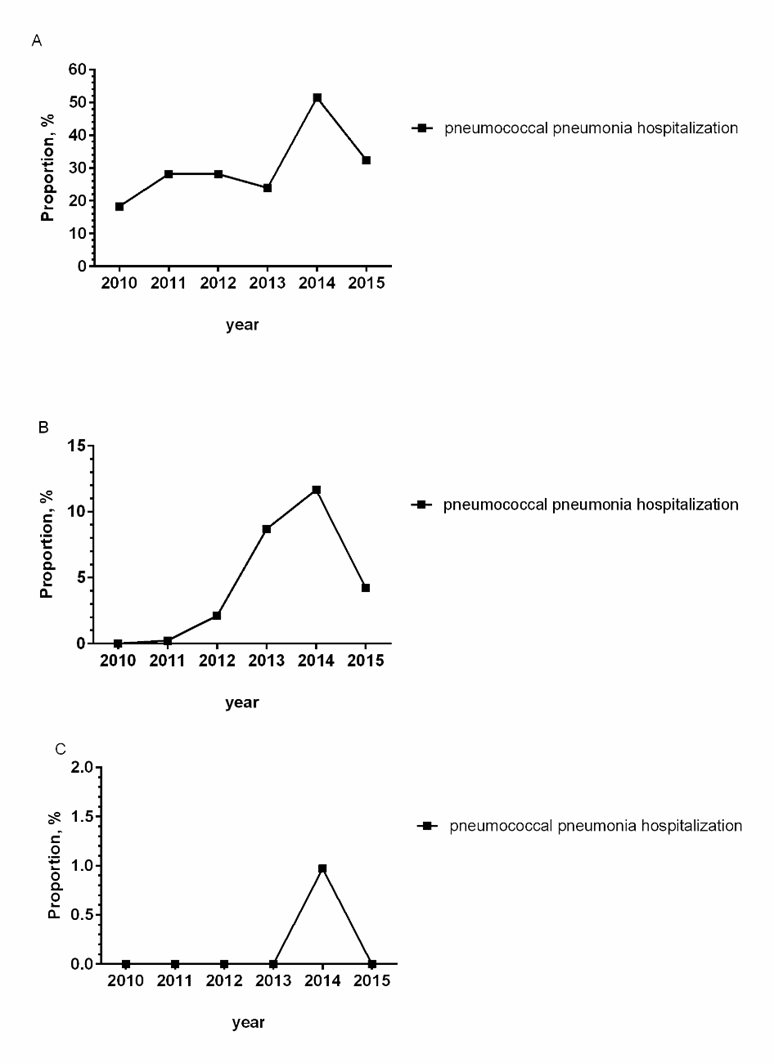
Supplementary Figure 1. Proportions of any type of pneumococcal vaccinations among children with all-cause pneumonia hospitalized from 2010-2015.** (A) 1 type of vaccine (B) 2 types of vaccine (C) 3 or more types of vaccine.
